# Vaccine-Induced Thrombotic Thrombocytopenia: A Case Report

**DOI:** 10.7759/cureus.23196

**Published:** 2022-03-15

**Authors:** Luciana Silva, Rita Pombal, Mariana Fidalgo, Andreia Freitas, Manuel Barbosa, Luisa Magalhães, Margarida Correia, João Valente

**Affiliations:** 1 Internal Medicine Department, Centro Hospitalar de Vila Nova de Gaia/Espinho, Vila Nova de Gaia, PRT; 2 Immunohemotherapy Department, Centro Hospitalar de Vila Nova de Gaia/Espinho, Vila Nova de Gaia, PRT; 3 Internal Medicine Department, Centro Hospitalar de Vila Nova de Gaia/Espinho, Vila Nova De Gaia, PRT

**Keywords:** covid-19, vaccine-induced thrombotic thrombocytopenia (vitt), janssen covid-19 vaccine, covid-19 vaccine, vitt covid-19

## Abstract

While the number of people who have been vaccinated against coronavirus disease 2019 (COVID-19) in Portugal keeps rising, the risk of complications, although rare, keeps rising too. We report a case of vaccine-induced thrombotic thrombocytopenia (VITT) in a 30-year-old previously healthy male after vaccination with Ad26.COV2.S. The patient presented to the emergency department (ED) with abdominal pain and headache. Laboratory tests revealed thrombocytopenia, high D-dimer levels, and fibrinogen consumption. Thoracoabdominal CT scan showed a thrombus in the portal mesenteric venous axis. A positive PF4 heparin enzyme-linked immunosorbent assay confirmed the VITT diagnosis, and the patient was started on intravenous immunoglobulin. Both clinical complaints and laboratory findings resolved within six days, and he was discharged to follow-up. This case shows that general symptoms after vaccination should not be depreciated, highlights the importance of early diagnosis and treatment, and raises new questions about the follow-up and further study of these patients.

## Introduction

The coronavirus disease 2019 (COVID-19) pandemic has a global impact affecting healthcare systems [1]. In Portugal, as much as the rate of infection was high, so was the rate of vaccination. On August 20, 2021, in Portugal, there were 1,014,632 confirmed cases, 44,916 active cases, and 17,622 deaths, corresponding to a 1.74% mortality rate [2]. The vaccination rate in Portugal in August 15 was 76% (7,791 486 people) with one dose and 66% fully vaccinated (6,760,777 people) [2].

The vaccination rate increased extremely fast around the world, and the first vaccine-induced thrombotic thrombocytopenia (VITT) cases associated with the ChAdOx1 nCoV-19 (AstraZeneca) vaccine were described in February 2021 [3]. In March 2021, cases associated with Ad26.COV2.S (Janssen) vaccine were reported [4].

In Portugal, until the end of October, nine VITT cases associated with the ChAdOx1 nCoV-19 vaccine and three cases with the Ad26.COV2.S vaccine among 16,246,592 vaccines administered were reported [5].

We reported a VITT case after the Ad26.COV2.S vaccine admitted in an intermediate care unit (IMU) in August 2021.

## Case presentation

A 30-year-old male patient presented in the emergency department (ED) with abdominal pain and headache. He had been vaccinated against COVID-19 with the Ad26.COV2.S vaccine 19 days prior. In the next two days, he complained of fatigue. Eight days later, he presented with fever and headache, for which he took ibuprofen, and on the 12th day, his main complaint was sudden-onset abdominal pain that would not resolve with medication. As symptoms persisted, he came to the ED.

**Figure 1:**
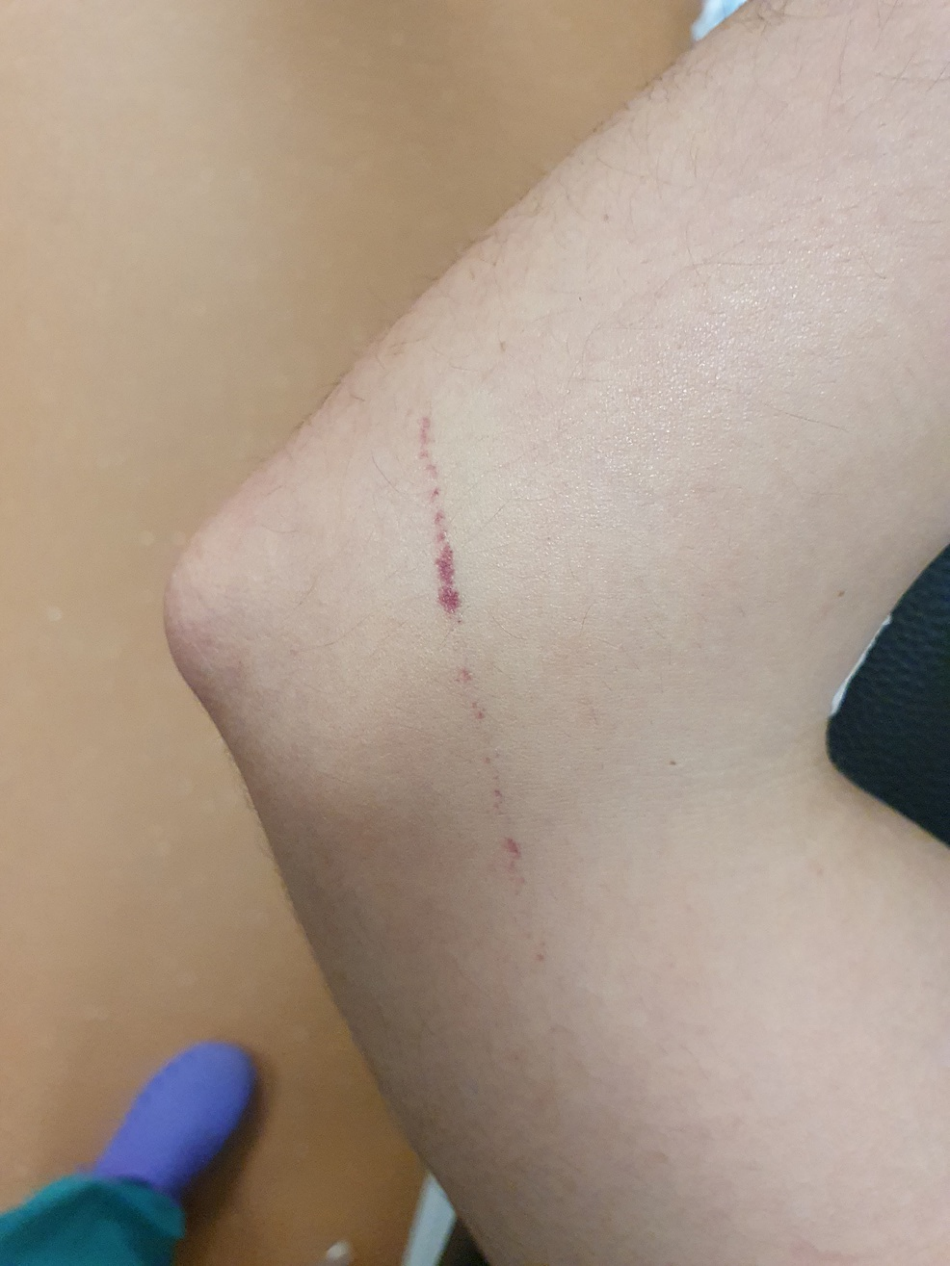
Petechiae on the patient’s right forearm

The patient had no past medical history and no chronic medication. He had no neurological deficit, fever, or respiratory insufficiency. His blood pressure and pulse were normal. Physical examination was unremarkable, except for petechiae on the right forearm (Figure 1).

The initial laboratory tests indicated thrombocytopenia (43,000 cells/mm^3^), low fibrinogen (93 mg/dL), prolonged prothrombin time (18.2 seconds) and activated partial thromboplastin time (56 seconds), and high D-dimer level (>20 µg/mL) (Table 1). Plasma creatinine, electrolytes, and liver enzymes were normal (Table 1). Reverse transcription PCR testing via nasopharyngeal swab returned negative for COVID-19.

**Table 1:** Test results at admission ALT: alanine aminotransferase, APTT: activated partial thromboplastin time, AST: aspartate aminotransferase, GGT: gamma-glutamyl transpeptidase, LDH: lactate dehydrogenase, NV: normal value, PT: prothrombin time, WBC: white blood cell

Laboratory test at admission	Results	NV
Hemoglobin	14.5 g/dL	13–18 g/dL
Platelet count	43,000 cells/mm^3^	150,000–450,000 cells/mm^3^
WBC count	7,150/uL	3,800–10,600/uL
PT	18.2 seconds	11.5–14.5 seconds
APTT	56 seconds	24–34 seconds
D-Dimer	>20 µg/mL	<0.5 µg/mL
Fibrinogen	93 mg/dL	200–400 mg/dL
Creatinine	0.93 mg/dL	0.67–1.17 mg/dL
Urea	35 mg/dL	13–43 mg/dL
Sodium	139 mmol/L	136–145 mmol/L
Potassium	4.1 mmol/L	3.5–5 mmol/L
Chloride	101.1 mmol/L	98–107 mmol/L
Total bilirubin	1.1 mg/dL	0.1–1.1 mg/dL
AST	23 U/L	4–33 U/L
ALT	44 U/L	4–50 U/L
Alkaline phosphatase	87 U/L	40–129 U/L
LDH	145 U/L	135–225 U/L
Albumin	4.6 g/dL	3.4–4.8 g/dL

**Figure 2:**
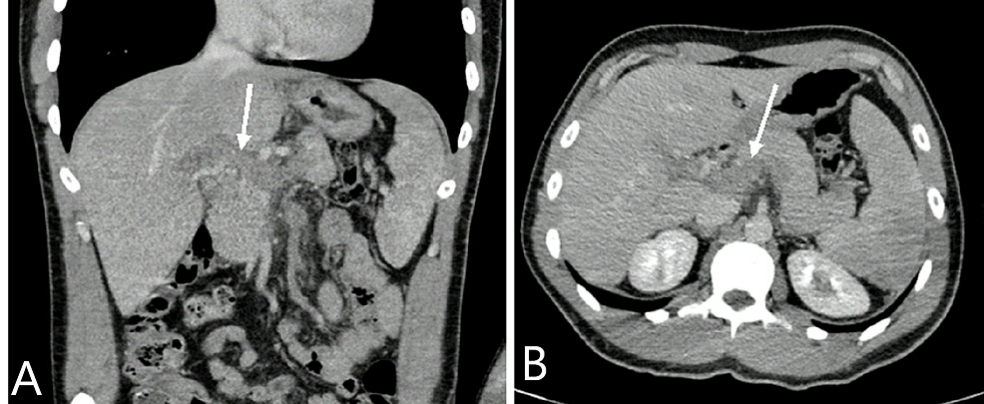
Thoracoabdominal CT scan showing portal mesenteric venous thrombosis (arrows) A: coronal view, B: axial view

A head CT scan was performed and was unremarkable. Thoracoabdominal CT scan showed a thrombus with complete occlusion of the portal mesenteric venous axis and cranial part of the superior mesenteric vein trunk (Figure 2).

VITT diagnosis was confirmed by a positive PF4 heparin enzyme-linked immunosorbent assay. We used the Asserachrom® HPIA kit (Diagnostica Stago, Asnières-sur-Seine, France) for the detection of anti-heparin/PF4 IgA, G, and M antibodies. The measurement is provided by the Multiskan^TM^ FC Microplate Photometer (Thermo Scientific^TM^, Waltham, MA, USA).

The patient was admitted to the intermediate care unit (IMU) and started on intravenous immunoglobulins 1 g/kg/day over two days plus four more days at the dose of 0.5 g/kg/day. He also received methylprednisolone 1 mg/kg/day and apixaban 5 mg bid since day 1, with anticoagulation therapy planned for three months.

The patient had a favorable clinical and analytical outcome, with progressive normalization of platelet count, D-dimer, and fibrinogen (Table 2). He was then discharged and reassessed as an outpatient (Table 3).

**Table 2:** Analytical evolution: platelet, D-dimer, and fibrinogen levels at each day APTT: activated partial thromboplastin time, NA: not available, PT: prothrombin time

Admission day	D0	D1	D2	D3	D4	D5	D6
Platelet count (cells/mm^3^)	43	44	89	119	151	181	187
PT (seconds)	18.2	18.1	16.2	NA	16.3	15.5	15.6
APTT (seconds)	56	34.9	29	NA	28.2	27.9	28.6
D-Dimer (µg/mL)	>20	>20	>20	10.59	5.58	4.55	3.45
Fibrinogen (mg/dL)	93	118	123	123	118	126	128

**Table 3:** Analytical evolution after discharge: platelet, D-dimer, and fibrinogen levels APTT: activated partial thromboplastin time, NA: not available, PT: prothrombin time

Day after discharge	D5	D13	D19	D21	One month	Two months
Platelet count (cells/mm^3^)	232	116	90	107	115	122
PT (seconds)	12.8	13.5	12.7	NA	12.9	12.5
APTT (seconds)	26.7	29.1	28.7	NA	30.4	31.7
D-Dimer (µg/mL)	1.95	NA	0.31	NA	0.45	0.26
Fibrinogen (mg/dL)	204	NA	348	NA	333	241

On the 19th day after discharge, the patient had an asymptomatic relapse, with thrombocytopenia (90,000/uL). He also had leukopenia (2,750/uL) and blasts in peripheral blood. A biopsy and medullary aspirate were performed and were normal, as was the karyotype. Peripheral blood immunophenotyping with flow cytometry was also performed and was normal too. Platelet counts improved. This was probably due to increased peripheral demand that lead to the proliferation of hematopoietic cells, with the presence of immature cells in peripheral blood. No other studies were performed, and no medication changes were made. The patient is still in regular follow-up.

## Discussion

The Ad26.COV2.S COVID-19 vaccine is a recombinant vaccine that uses replication-incompetent human adenovirus 26 (Ad26) as a vector to express the SARS-CoV-2 Spike (S) protein [6].

The incidence of VITT is unknown but appears to be very rare [3]. The highest incidence was reported in Norway, one in 26,000 [7]. Nevertheless, it’s important to recognize VITT symptoms due to their potential severity.

The VITT cases reported to date usually occur in women younger than 40 years old without any previous heparin exposure [4]. Thrombosis most frequently occurs in the cerebral venous sinus, with the remaining cases affecting a range of territories such as the splanchnic system, heart, lungs, or limbs. Prognosis improves with earlier recognition, but fatality rates can be high. In a series of 220 individuals with definite or probable VITT, the mortality rate was 22% [8]. Treatment includes non-heparin-based anticoagulation (mostly with a direct oral anticoagulation) and high-dose intravenous immunoglobulin with plasma exchange and immunosuppressants reserved for severe or refractory disease. Platelet transfusion should be reserved for critical bleeding [9,10].

VITT’s natural history is still not well understood, with little published evidence available. Most patients still have thrombocytopenia and PF4 antibodies after discharge from the hospital. Recommendations from the Natural Institute of Health and Care Excellence advise continued monitoring until antibodies are no longer detected. The benefit of prolonged corticotherapy is unknown [10].

## Conclusions

This case has some atypical details, as it refers to a male patient, with thrombosis occurring in the portal mesenteric and superior mesenteric vein and recurrence of thrombocytopenia after discharge.

It also shows that general symptoms after vaccination should not be depreciated. They may be the only symptoms of a life-threatening situation. It highlights the importance of early diagnosis and treatment in a patient with thrombocytopenia less than one month after a recombinant vaccine, but it also raises new questions about follow-up. It is important that physicians report similar cases in order to improve the management of these patients.
